# Adult patients with alopecia areata report a significantly better medication adherence compared to those with atopic dermatitis: Results from a large cross-sectional cohort study

**DOI:** 10.1016/j.jdin.2024.03.026

**Published:** 2024-04-16

**Authors:** Mischa J. Mallbris, Lea Krog Nymand, Yuki Maria Fukuda Andersen, Alexander Egeberg

**Affiliations:** a Park Tudor School, Indianapolis, Indiana; b Department of Dermatology, Bispebjerg Hospital, Copenhagen, Denmark; c Faculty of Health and Medical Sciences, Department of Clinical Medicine, University of Copenhagen, Copenhagen, Denmark

**Keywords:** alopecia areata, atopic dermatitis, MARS-5, treatment behavior

## Abstract

**Background:**

Alopecia areata (AA) and atopic dermatitis (AD) are chronic skin diseases where the suboptimal medication adherence (MA) may result in poor clinical outcomes.

**Objective:**

To assess the impact of AA on MA among adults compared to AD.

**Methods:**

Patient reported MA of adults with AA were compared with AD. Patients were identified from the Danish Skin Cohort, a nationwide prospective cohort of dermatological patients in Denmark. We used the Medication Adherence Report Scale- 5, a self-reporting questionnaire, to assess MA. Demographic and disease characteristics were collected. Logistic regression was conducted.

**Results:**

Patients with AA reported higher MA than AD (mean 21.81 vs 18.29). Logistic regression analyses showed AA diagnosis had a statistically significant positive effect on MA (odds ratio = 3.94, 95% CI 2.01-8.89). Men reported significantly higher MA (odds ratio = 1.49, 95% CI 1.14-1.94). Current disease severity did not impact MA.

**Limitations:**

Data were self-reported by patients. Data regarding the specific treatment undergone by patients were not available.

**Conclusion:**

Patients with AA have significantly higher MA compared to patients with AD. The stability of AA patients’ symptoms may lead to higher MA due to a desire for disease control. Conversely, the sporadicity of AD symptoms could negatively affect adherence, causing fluctuations in medication use.

**Capsule Summary**

• Medical adherence is vital to disease management of skin disorders. We provide insight by observing 2 common dermatological diseases and their associations with medical adherence.
• The results indicate the different nature of alopecia areata and atopic dermatitis, and a need for unique approaches when tackling treatment management for each diseases.

## Introduction

Chronic skin disorders are among leading causes of nonfatal disease burden worldwide,^1^ and account for a significant proportion of total healthcare utilization. The optimal therapeutic management of chronic skin disorders depends critically on medication adherence (MA). Poor MA among chronic diseases is a complex and critical challenge in today's health care system. While poor MA poses a significant financial burden, it may furthermore have consequences on the care management of patients, with unnecessary switch of treatments and dose escalations.^2^ The main factors impacting MA among patients with dermatological disorders include lack of patient training about the disease or the treatment, lack of belief in the treatment, poor prior experience, side effects or fear of it, and cost of medication among others.^3^

In managing patient care, it is important to understand how different chronic skin conditions affect MA. Alopecia areata (AA) and atopic dermatitis (AD) are common immune-mediated skin diseases which affect nearly 2% and 10% of the general population at some point in their lifetime, respectively.^4^^,^^5^ AA is characterized by nonscarring hair loss with preservation of affected hair follicles,^5^ whereas AD is characterized by intense itch, inflamed lesions caused by the underlying inflammation, and impairment of the skin barrier.^4^, ^5^, ^6^ Both diseases are associated with a profound negative impact on quality of life,^7^, ^8^, ^9^, ^10^, ^11^ and a successful treatment management of AA and AD has been shown to significantly improve quality of life.^12^^,^^13^ To achieve optimal outcomes for patients, a better understanding of how factors such as disease burden and patient characteristics in specific patient populations can impact the individual patients’ MA is warranted.

The aim of the current study was to assess the impact of adult AA vs AD on MA. Also, we aimed to identify demographic and disease characteristics that may play a role in MA among adults with AA.

## Materials and Methods

All study participants provided a written informed consent before participating. The study was registered according to the Danish Data-protection Agency (Videncenter for Dataanmeldelser, ref. P-2021-386). This constitutes the necessary legal requirements, and ethical approval is not required for this type of study in Denmark.

### Data sources and study population

The Danish Skin Cohort served as the data source for the 2 patient cohorts included in this study. The data collection method and the initial characterization of the patient populations are previously described in detail.^14^ Briefly, the Danish Skin Cohort was established in 2018 as a prospective inception cohort of dermatological patients in Denmark and follow-up data were collected and added in 2020, 2022, and at least once a year hereafter.^14^ Patients with at least 1 diagnostic code for AA verified by a dermatologist after their 18th birthday were identified as the primary study population. Patients with AD (at least 1 dermatologist-verified diagnostic code for AD after their 18th birthday) served as the control group, as both patient groups suffer from chronic skin diseases and were most comparable in terms of patient characteristics. All identified patients received an invitation by secure electronic governmental mail and answered the survey electronically. Study data were collected and managed using REDCap (Research Electronic Data Capture)^15^^,^^16^ a secure, web-based software platform designed to support data capture for research studies.

### Outcome measure

As the primary outcome measure, we used the Medication Adherence Report Scale (MARS-5), a validated measure for MA.^17^ Here the patients were asked how often they do 5 different behaviors toward their treatment utilization, for example, “I take less than instructed” and have the answers: “Very often,” “Often,” “Sometimes,” “Rarely,” and “Never”. The answers “Rarely” and “Never” were seen as high adherence.

### Covariates

We collected information regarding participants’ age, gender, height, weight, ethnicity, Fitzpatrick skin type,^18^ and natural hair color. Additionally, we asked participants to answer questions regarding levels of physical activity and a rating of general health on a scale from 0 to 100, where 0 is worst possible health and 100 is best possible health. Furthermore, disease specific data regarding onset of disease, disease activity and severity, prescribed treatment and Dermatology Life Quality Index,^19^ were collected.

### Statistical analysis

The patient populations with AD and AA were characterized using descriptive statistics including mean, standard deviation, median, and interquartile ranges, according to data distribution. Frequencies were calculated for categorical variables including percentages of the primary endpoints. Odds ratios (ORs) for the primary outcome were estimated using logistic regression models. The selected covariates in the adjusted models were gender, age and disease severity. Disease severity was defined as current severity rated on a numeric rating scale from 0 to 10 where 10 was “worst possible AA.” Predictors for MA were further estimated in multiple regression models. Statistical analyses were conducted using RProject software, version 4.1.2 (R Foundation for Statistical Computing) with packages “Tableone.”

## Results

**Fig 1:**
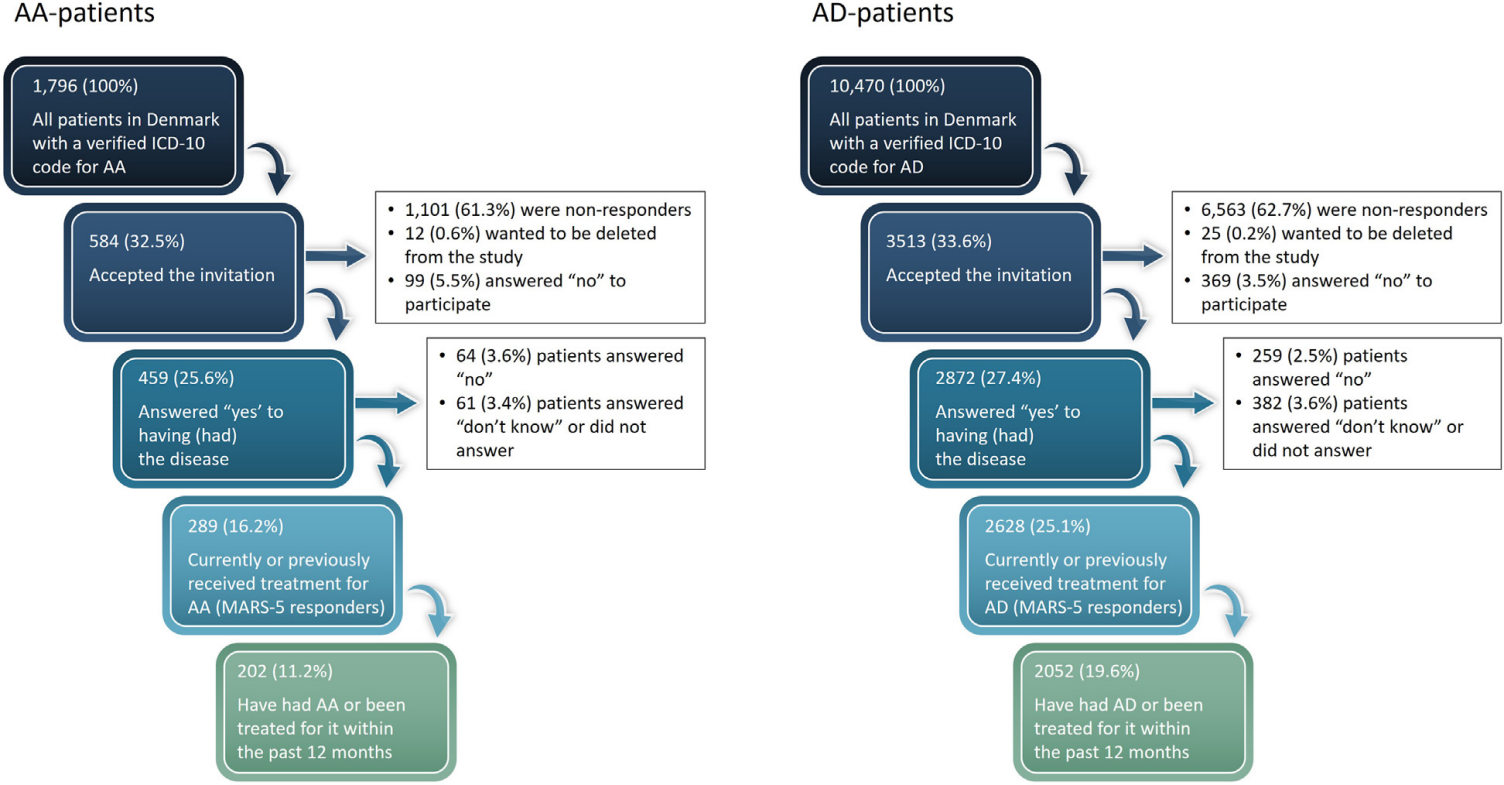
Flowchart for the patient selection process.

In total, 3331 adult patients (≥18 years) with confirmed skin diagnosis were identified from Danish Skin Cohort with either a diagnosis of AA (*N* = 459) or AD (*N* = 2872). The patient selection process is visualized in the flowchart in Fig 1.

The overall baseline characteristics of groups are presented in Table I. At time of the present survey, patients with AD were younger than those with AA, (mean 47.0 vs 54.6 years old). In general, >70% of the patients, in either group, were females. Both groups reported similar levels of physical activity (Table I).

**Table I:** Baseline characteristic

	Alopecia areata	Atopic dermatitis
	*N* = 459	*N* = 2872
Age, y		
Mean (SD)	54.6 (13.8)	47.0 (13.5)
Median (IQR)	55 (46-65)	46 (37-56)
Sex, *n* (%)		
Female	330 (71.9)	2027 (70.6)
Male	129 (28.1)	845 (29.4)
Physical activity, *n* (%)		
Sedentary	79 (17.2)	548 (19.1)
Moderate	274 (59.7)	1619 (56.4)
Vigorous	100 (21.8)	653 (22.7)
Athletic	<5 (<1.1)	26 (0.9)
Unknown/missing	<6 (<1.3)	26 (0.9)
Fitzpatrick skin type, *n* (%)		
1	20 (4.4)	127 (4.4)
2	92 (20.0)	848 (29.5)
3	266 (58.0)	1529 (53.2)
4	75 (16.3)	352 (12.3)
5	<5 (<1.1)	9 (0.3)
6	<5 (<1.1)	<5 (<1.2)
Unknown/missing	0	<7 (0.2)
Natural hair color		
Blonde	117 (25.5)	803 (28.0)
Light brown/reddish	92 (20.0)	692 (24.1)
Brown	154 (33.6)	967 (33.7)
Dark brown or black	85 (18.5)	399 (13.9)
Never had hair	5 (1.1)	0
Unknown/missing	6 (1.3)	11 (0.4)
Patient rated overall health (NRS 0-100)		
Mean (SD)	71.6 (22.1)	72.7 (19.8)
Median (IQR)	78 (60-87)	78 (65-86.5)

*IQR*, Interquartile range; *NRS*, numeric rating scale; *SD*, standard deviation.

Table II provides an overview of patients reported disease characteristics. Additionally, a total of 766 patients (*N* = 125 with AA, *N* = 641 with AD) who did not actively confirm their skin disease diagnosis were included in the analysis.

**Table II:** Alopecia areata disease specific characteristics

AA questionnaire	*n* (%)	AD questionnaire	*n* (%)
	459 (100)		2872 (100)
Age at AA onset		Age at AD onset	
0-10 y old	30 (6.5)	0-5 y old	1646 (57.3)
11-18 y old	45 (9.8)	6-18 y old	777 (27.1)
19-45 y old	225 (49.0)	>18 y old	425 (14.8)
≥46	160 (34.6)		
Missing	0	Missing	25 (0.9)
Disease duration (*n* = 353)		Disease Duration (*n* = 1348)	
Median (IQR)	14 (8-22)	Median (IQR)	41 (29-50)
Did you have AA or received treatment for AA within the past 12 mo?		Did you have AD or received treatment within the past 12 mo?	
Yes	202 (44.0)	Yes	2052 (63.1)
No	257 (56.0)	No	809 (24.9)
Unknown/missing	0	Unknown/missing	11 (0.4)
Current severity of AA (NRS 0-10)		Current severity of AD (NRS 0-10)	
Median (IQR)	8 (4-10)	Median (IQR)	3 (2-6)
Number of flare ups (within past 12 mo)		Number of flare ups within the past 12 mo	
Median (IQR)	8 (4-10)	Median (IQR)	6 (3-12)
Rate how your AA has evolved during the last 12 mo		Rate how your AD has evolved during the past 12 mo	
Strongly aggravated	17 (3.7)	Strongly aggravated	30 (1.0)
Aggravated	29 (6.3)	Aggravated	300 (10.4)
Unchanged	113 (24.6)	Unchanged	1052 (36.6)
A little better	22 (4.8)	A little better	286 (10.0)
A lot better	9 (2.0)	A lot better	291 (10.1)
Completely gone	12 (2.6)	Completely gone	49 (1.7)
No AA within the past 12 mo	257 (56.0)	Missing/no AD within the past 12 mo	864 (30.1)
Have you received prescribed treatment for AA?		Have you received prescribed treatment for AD?	
Yes, currently receiving treatment	43 (9.4)	Yes, currently receiving treatment	1795 (62.5)
Yes, previously received treatment	246 (53.6)	Yes, previously received treatment	833 (29.0)
No	165 (35.9)	No	160 (5.6)
Missing	5 (1.1)	Unknown/missing	84 (3.0)
DLQI		DLQI	
Median (IQR)	1 (0-4)	Median (IQR)	3 (1-7)

*AA*, Alopecia areata; *AD*, atopic dermatitis; *IQR*, interquartile range; *NRS*, numeric rating scale; *DLQI*, Dermatology Life Quality Index.

**Fig 2:**
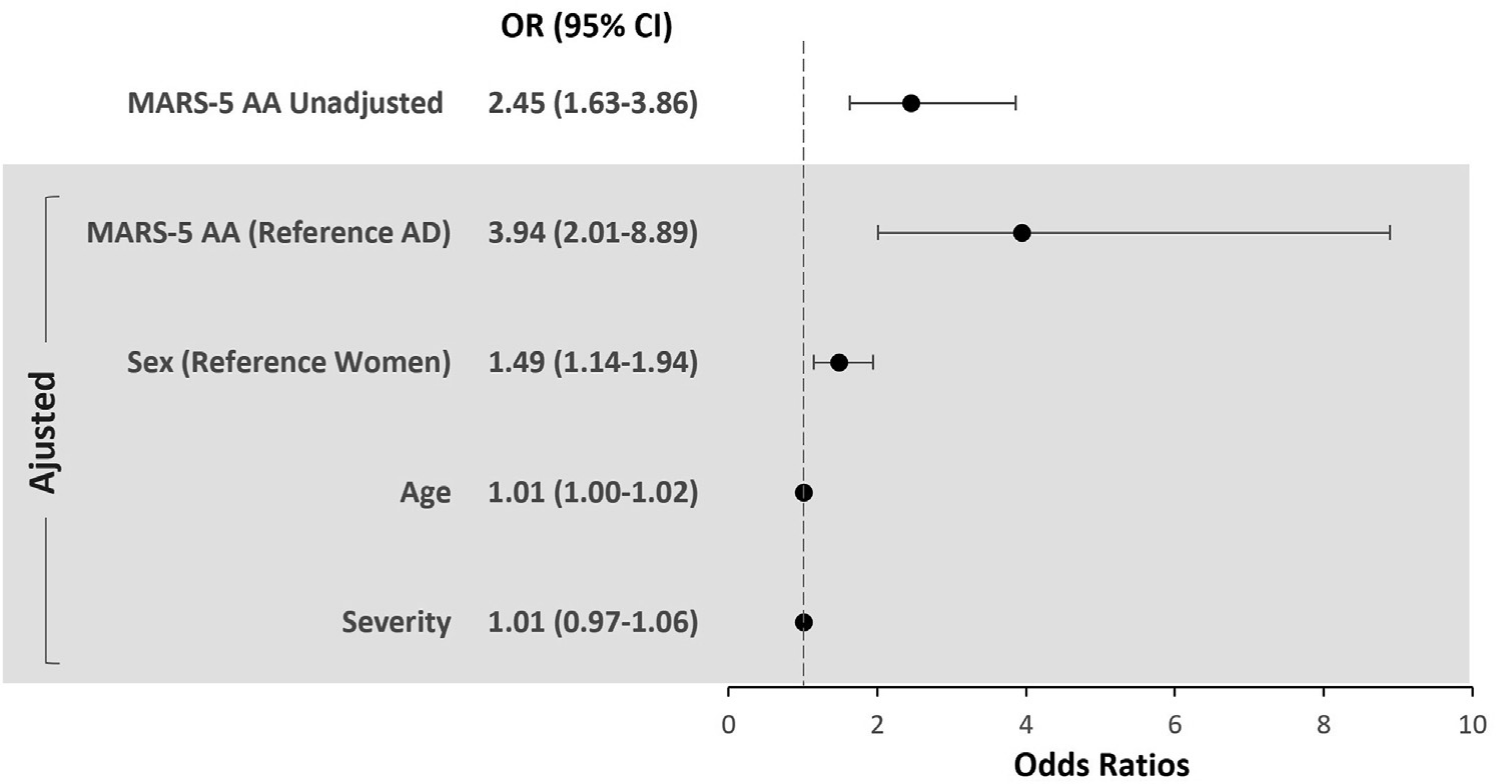
Medical adherence results.

As expected, most adults with AD had an onset of their disease at a younger age (84.4% with an onset <18 years old) with a median (interquartile range) age of 46 (37-56) years, compared to adults with AA (83.6% reported an onset >18 years old) with a median (interquartile range) age of 55 (46-65) years. Therefore, patients with AD reported longer disease duration. A higher percentage of patients with AD reported receiving current treatment (62.5%) or previously received treatment (29.0%) for their skin disease compared with AA (9.4% and 53.6%). Patients with AA reported a higher ongoing severity of their disease activity (medical numeric rating scale = 8 for AA vs numeric rating scale = 3 for AD). On the other hand, patients with AD reported more flare ups within the past 12 months compared to patients with AA who reported having a more stable disease. The MA results are shown in Table III and Fig 2.

**Table III:** Medication Adherence Report Scale-5

MARS-5	AA *n* = 289	AD
		*n* = 2628
MARS-5 score (range 5-25) mean (SD)	21.8 (4.6)	18.3 (4.6)
Median (IQR)	24 (20-25)	19 (15-22)
I take less than instructed		
Very often	17 (5.9)	187 (7.1)
Often	7 (2.4)	279 (10.6)
Sometimes	18 (6.2)	718 (27.3)
Rarely	41 (14.2)	616 (23.4)
Never	193 (66.8)	689 (26.2)
Missing	13 (4.5)	139 (5.3)
I stop taking it for a while		
Very often	30 (10.4)	483 (18.4)
Often	14 (4.8)	436 (16.6)
Sometimes	15 (5.2)	594 (22.6)
Rarely	34 (11.8)	377 (14.3)
Never	183 (63.3)	599 (22.8)
Missing	13 (4.5)	139 (5.3)
I miss out a dose		
Very often	14 (4.8)	102 (3.9)
Often	6 (2.1)	160 (6.1)
Sometimes	26 (9.0)	526 (20.0)
Rarely	65 (22.5)	874 (33.3)
Never	164 (56.7)	826 (31.4)
Missing	14 (4.8)	140 (5.3)
I alter the dose		
Very often	13 (4.5)	146 (5.6)
Often	2 (0.7)	201 (7.6)
Sometimes	18 (6.2)	534 (20.3)
Rarely	30 (10.4)	573 (21.8)
Never	212 (73.4)	1034 (39.3)
Missing	14 (4.8)	140 (5.3)
I forget to take it		
Very often	13 (4.5)	82 (3.1)
Often	6 (2.1)	126 (4.8)
Sometimes	20 (6.9)	492 (18.7)
Rarely	69 (23.9)	918 (34.9)
Never	167 (57.8)	870 (33.1)
Missing	14 (4.8)	140 (5.3)

*AA*, Alopecia areata; *AD*, atopic dermatitis; *MARS-5*, The five-item Medication Adherence Report Scale.

In total, 63% (289/459) adults with AA, and 91% (2628/2872) adults with AD completed the MARS-5 questionnaire. Overall, patients with AA reported significantly higher MA than those with AD (mean (standard deviation) MARS-5 score 21.8 [4.6] vs 18.3 [4.6]). The comparison analysis between AA and AD showed a statistically significant difference and a higher level of MA among adults with AA. The adjusted OR showed almost 4 times higher MA among adults with AA compared to those with AD (OR = 3.94, 95% CI 2.01-8.89, *P* < .001). Surprisingly men reported a significantly higher MA than women (OR = 1.49, 95% CI 1.14-1.94, *P* < .005). Also, unexpectedly, disease severity did not impact the MA (*P* = .63).

The analysis was also done for only patients with AA and AD currently receiving treatment and the results were similar regarding MARS-5 (data not shown).

## Discussion

The aim of this study was to investigate the impact of AA on MA of adults in comparison to MA of patients with AD. Indeed, this study supports the notion that different skin diseases have a different impact on MA of patients, as previously described by Feldman et al in patients with AD compared to those with psoriasis or hand dermatitis (reference). Additionally, the study results indicate that patients with AA have a significantly higher MA than patients with AD, regardless of disease severity.

Previous studies have compared the burden of illness and the prescription usage of patients with AA and those with AD,^20^ yet no study has investigated the impact of AA vs AD on the MA in adults.^21^

Interestingly, patients with AA showed a tendency of better adherence to prescribed therapies than patients with AD, despite the lack of effective treatments for AA until very recently. This finding possibly reflects the significant burden of disease and high desire to achieve improvement in symptoms among patients with AA. It is well established that AA may significantly affect patients’ self-esteem and quality of life and the unpredictable disease course and fear of relapse may lead to a stronger motivation to follow therapies as prescribed. On the other hand, patients with AD may experience frequent fluctuations in their symptoms, leading to periods with alternating good and bad adherence.

To our surprise, women were found to have a significantly lower MA than men. While studies assessing the impact of sex on MA among dermatological disorders are scarce, aligned with our empirical expectation, female patients with dermatitis herpetiformis had a better MA to a gluten-free diet compared to men.^22^ There could be several potential root causes for this finding, as demonstrated by studies in other therapeutic areas and regions reporting an association with gender differences in MA. One study conducted in the US adult population showed a lower MA among women due to costs related to medical care.^23^ While Denmark offers a tax-supported national medical coverage which includes largely free primary and specialist care, a capped copayment for drugs filled at community pharmacies is applied, which may contribute to a lower MA among women aligned with findings in the above-mentioned report.^24^ It is conceivable that cost of copayment of medications might have influenced the MA leading to the observed gender differences, as previously shown in a Japanese study.^25^ Indeed, we found significant socioeconomic difference between male and female patients with AD (data not shown). However, we did not find significant differences in personal income between male and female patients with AA in our cohort, indicating that the difference is not explained by financial factors alone. Other factors that have been discussed in the literature are fear of transmission of medicines to the breast milk while women are pregnant or nursing.^26^ Again, we have not studied this factor as a potential bias in our population. Furthermore, it is unclear if social desirability bias could have influenced men to self-report a better MA pattern than women. Nevertheless, independent of the root causes, the results highlight a need for dermatologists to consider gender differences in MA in their efforts to enable patients to effectively adhere to their treatment.

In disease management, understanding the nature of chronic skin diseases and their impact on MA is critical for patient care. While we have not studied how to overcome these differences, other studies have shown and recommended how to improve the MA in patients with AD^3^^,^^27^^,^^28^; whereas such studies and recommendations are lacking in AA care management. It would be interesting to evaluate if there is an increased use of these tools to enhance adherence and outcome for patients with AA in general compared to patients with AD. In this context, prior studies suggested the development of clearly written treatment plans to help simplify the treatment regiments for the patients with AD will increase the MA.^3^ Additional tactics included reducing the time intervals between the follow-up visits by increasing patients and health care provider remote/virtual contacts^21^^,^^29^ as well as increasing the level of patient’s understanding of their condition, by strengthening patient education.^3^ Others have demonstrated an increased MA by using digital interventions including using mobile app, treatment reminders, and lifestyle coaching.^30^

While, in the current study, we tried to limit biases through adjustment for some baseline characteristics, factors including use of tactics to enhance MA, as mentioned above, and differences in socioeconomic status and social support that could impact MA, were not adjusted for. Therefore, further studies controlling for these factors/biases are necessary to fully understand and verify the true MA differences between these 2 patient groups. The ultimate assessment would be in the form of a longitudinal prospective study to determine whether the MA of patients with AA and AD increase or decrease as disease management continues over time.

## Conclusion

In this cross-sectional survey, we observed that patients with AA have a higher MA than patients with AD, possibly reflecting an unrecognized burden of disease or unmet medical needs in patients with AA. Individual patient characteristics should be incorporated in clinical decision making and assessment of therapeutic goals.

## Conflicts of interest

Dr Andersen has received speaker honoraria from Eli Lilly and Company. Dr Egeberg has received honoraria as consultant and/or speaker from Amgen, AbbVie, Almirall, Leo Pharma, Zuellig Pharma Ltd., Galápagos NV, Sun Pharmaceuticals, Samsung Bioepis Co, Ltd, Pfizer, Eli Lilly and Company, Novartis, Union Therapeutics, Galderma, Dermavant, UCB, Mylan, Bristol-Myers Squibb, McNeil Consumer Healthcare, Horizon Therapeutics, Boehringer Ingelheim, and Janssen Pharmaceuticals. Authors Mallbris and Nymand have no conflicts of interest to declare.
